# Presymptomatic Screening for Risks to Children’s Mental Health: Ethical Considerations from a European Focus Group Study with Mental Health Professionals

**DOI:** 10.1007/s11673-025-10473-0

**Published:** 2025-09-15

**Authors:** Sammie N. G. Jansen, Bob C. Mulder, Alexandra E. Boekhold

**Affiliations:** 1 Department of Social Sciences, Chair Group Philosophy, Wageningen University & Research, Hollandseweg 1, Wageningen, 6706 KN The Netherlands; 2 Centre for Sustainability, Environment and Health, National Institute for Public Health and the Environment, RIVM, P.O. Box 1, Bilthoven, 3720 BA The Netherlands; 3 Department of Social Sciences, Chairgroup Strategic Communication, Wageningen University & Research, Hollandseweg 1, Wageningen, 6706 KN The Netherlands

**Keywords:** Presymptomatic screening, Ethics, Child mental health, Stakeholders, Focus group study

## Abstract

The development of presymptomatic screening for risks to children’s mental health holds the promise to prevent or reduce the burden of mental disorders by enabling timely preventive actions. However, such screening programmes also raise ethical concerns related to false positive results, increased anxiety, harmful effects on a child’s sense of self, and stigmatization. Stakeholders can provide valuable insights into these ethical concerns from their engagement with practice. Therefore, in this study we conducted six focus group discussions with professionals in the child mental health domain (in clinical, educational, or policy settings) to investigate their views on presymptomatic screening and identify ethical considerations. The discussions took place in six European countries. Three main themes were identified: 1) Promises and concerns about screening for risks to children’s mental health, 2) Additional considerations about biomarker screening, and 3) Implications for healthcare systems and society. Ethical considerations included the benefits of screening outweighing the harms, informed and autonomous decision-making, the actionability of screening outcomes, stigmatization, and medicalization. Our findings underscore the importance of exercising caution in the development and implementation of presymptomatic screening for risks to children’s mental health. Implications for practice and future research are discussed.

## Background

Presymptomatic screening for risks to children’s mental health aims to identify environmental, social, and biological risk factors and the individuals or populations at risk of developing mental health disorders before clinical symptoms emerge. Such screening programmes promise to prevent or reduce the burden of disease by taking timely preventive actions, being beneficial to both clinical and public health settings (Kim et al. 2022; Wakschlag et al. 2019). When writing “screening,” “screening programme,” or “screening practice” in this article, we refer to presymptomatic screening, unless stated otherwise.

Within bioethics, the ethics of presymptomatic screening for mental disorders is primarily focused on genetics (Chadwick 1999; Fulda and Lykens 2006; Mezinska et al. 2021; Nuffield Council on Bioethics 1993). However, research suggests that genetic factors alone only account for a small portion of the risk of developing mental diseases, highlighting the need to consider non-genetic factors as well (Arango et al. 2021; Hollander et al. 2020). Exposome research addresses this need by examining the complex interplay between biomarkers and physical and social environmental exposures over the lifetime (Kamp et al. 2022; Wild 2005). Exposome research can also take into account long term exposures and risk factors from early childhood to disease onset in youth and later life. As our understanding of the exposome continues to evolve, it is essential to address the ethical and societal implications of presymptomatic screening practices that incorporate non-genetic biomarkers and environmental factors. After all, the ethical and societal implications of presymptomatic screening may also be relevant to application of knowledge about the exposome. Therefore, this study aims to examine stakeholder views of child mental health professionals on such screening practices and identify ethical considerations, specifically in relation to mental health.

Several benefits of screening have been proposed. First, implementing programmes screening for risks to children’s mental health is proposed to increase health equity by identifying and enabling support for those who are most in need and can benefit most from preventive interventions (Glenn 2019). Furthermore, the presymptomatic detection of risk factors is proposed to allow families to plan for the future, adapt their life plans, and arrange the support their child may need in the future in a timely manner (Ahlgrim et al. 2019; Glenn 2019; Spriggs et al. 2008; Hoge and Appelbaum 2012).

Screening programmes can operate on different levels, targeting different age groups and developmental phases, risk factors, and disorders, and use different tools and technologies. Community-level screening focuses on population risk factors such as poverty or lack of access to healthcare; group- and individual-level screening focus on external risk factors such as maternal depression or catastrophic events; and screening for individual characteristics focuses on detecting personal risk factors such as early symptoms, behaviours, or biomarkers (National Research Council and Institute of Medicine. et al. 2009). Screening programmes for risks to children’s mental health often assess risk factors for a broad range of mental health issues (Feeney-Kettler et al. 2010), but there are also programmes that screen for specific disorders such as ADHD or depression (Goodman et al. 2000; Thombs et al. 2012). Various tools are developed or under development for presymptomatic mental health screening, varying from more established tools such as questionnaires and behavioural assessments to emerging tools such as machine learning technologies and biomarker tests (Feeney-Kettler et al. 2010; De Sousa Maciel et al. 2023; Tate et al. 2020).

The implementation of presymptomatic screening programmes, however, raises ethical concerns (Vanderschaeghe et al. 2018; Jansen et al. 2024). In offering a screening programme, difficulties with obtaining informed consent can occur and create tensions between parents’ rights to know their child’s susceptibilities, and a child’s right to know (or not know) their susceptibilities (Hall et al. 2014; Hoge and Appelbaum 2012). The resulting risk information can have harmful effects on the child and caregivers as it may affect the child’s identity, including how parents, family, and educators see and raise the child. By itself, this may become a barrier to healthy development and the child’s social and educational opportunities, possibly having lifelong effects (Singh and Rose 2009; Specker and Schermer 2017). Other concerns for detecting risk factors arise from the high complexity of human behaviour and mental health development, as a bias towards “simple” (biological) explanations and predictions for mental disorders can lead to high rates of false positive screening results. Furthermore, targeting personal risks arguably leads to an excessive focus on individual medical interventions rather than addressing social and environmental risk factors and causes, especially for biomarker-based screening programmes (Singh and Rose 2009; Specker and Schermer 2017; Thomas 2015). Finally, stigmatizing and discriminating effects are feared both for high-risk individuals and high-risk groups that often are already marginalized, for example, ethnic minority groups or groups of low socio-economic position, possibly increasing the harmful effects described above (Ahlgrim et al. 2019; Gershon 2013; Salamanca-Buentello et al. 2020; Singh and Rose 2009; Specker and Schermer 2017; Spriggs et al. 2008).

To gain a comprehensive understanding of these ethical dimensions of (future) screening practices it is crucial to include stakeholder perspectives. Various stakeholders have an impact on, and are impacted by, screening practices and may bring forward different ethical considerations. Their experience in practice provides them with insights into what is ethically relevant, because they have experiential moral knowledge (Molewijk et al. 2004; Widdershoven et al. 2009). So far, however, the ethics literature in this domain knows a scarcity of empirical studies that include stakeholder voices. This paper focuses on one specific stakeholder group, namely professionals working in the child and/or adolescent mental health domain (in clinical, educational, or policy settings). These professionals have an important stake in child mental health practices as they must consider what is best for individual children or groups of children and what methods to apply in preventing mental disorders. We deem the perspectives of these professionals especially relevant as they are either involved in the contexts where (presymptomatic) signs of mental health problems are detected and treated or involved in the policy contexts that shape screening practices. Therefore, we aim to investigate their views on presymptomatic screening and identify ethical considerations. Exploring these perspectives allows us to contribute empirical-normative considerations to the bioethics debate on presymptomatic screening.

## Methods

### Study Design

Focus groups are structured group discussions suitable for studying relevant experiences and related thoughts and feelings on the topic under discussion (Kitzinger 1995). The focus group methodology was chosen for its eligibility to study what opinions, views, and values exist within certain groups about a topic or issue. The participants were invited to explicate their perspectives, i.e. experiential knowledge, on screening for risks to children’s mental health and reflect on implications for children, their direct environment, and the wider society. An important feature of the methodology is that participants can respond directly to each other, creating series of associations, personal experiences, norms, and values (Kitzinger 1995). Alternating between individual thoughts and experiences and collective exchanges in which one participant’s experiences and thoughts trigger responses and insights in other participants allows for assessing the plausibility of the expressed views (Boenink et al. 2018; Felt et al. 2014). To avoid influencing participants’ responses, we refrained from providing examples of screening practices or mental health disorders (although participants were free to discuss those when brought up) and from directing the discussions substantively. The study was designed, performed, and analysed in accordance with Krueger and Casey’s guide for focus group research (2015).

### Sampling and Recruitment

Ethical approval was granted by the Social Sciences Ethical Committee of the Wageningen University and Research under file number 2022–108-Jansen. All participants provided verbal informed consent for participation, audio recording of the discussion, and anonymized publication of the results. Participants did not receive monetary compensation for their participation.

Within the context of the Equal-life project, focus groups with five to six participants were held in six European countries between September 2022 and April 2023. These countries were North Macedonia (Skopje), Slovenia (Ljubljana), Italy (Milan), Germany (Bremen), the Netherlands (Bilthoven), and Sweden (Stockholm). Using a purposive sampling approach, participants (thirty-four in total) were identified through the stakeholder network of the Equal-life project and were asked to recommend professionals from their network (snowball method; Krueger and Casey 2015). Eligibility criteria for participants were working within the child and/or adolescent mental health domain, in clinical or educational settings, or in the child mental health policy domain. These professionals are important stakeholders in the field of prevention of mental disorders, engaging with screening practices and outcomes at different levels. Therefore, no direct experience with the practice of screening for mental health was required. Participants received a short information letter stating the topic of the focus group study, as well as its relationship to the Equal-life project. Overall, however, participants were provided with minimal information to prevent unintended shaping of participants’ own thoughts and views (Boenink et al. 2018).

### Focus Group Discussions

The focus groups were moderated in the local language unless consultation with local colleagues and the participants indicated that the session could equally well be moderated in English. In the latter case, the preference was given to the first author moderating in English. This applied to the focus groups in Ljubljana and Stockholm. A topic list was developed through discussions between all authors, the project coordinator, and the leader of the project’s stakeholder engagement work package. The topic list contained explanations of the aims and methodology for the discussion moderator, the main topics, proposed follow-up questions, and time indications for each part. The topic list enabled the same discussion structure across focus group sessions, see appendix A. The moderator remained neutral by refraining from the discussion and only facilitated the session by asking questions. The focus groups were held in person, except two occasions where one participant joined online, discussing the topics with the other participants who were physically present in the room. The focus groups lasted two hours.

The sessions started with a brief introduction by the moderator on the topic and definitions under discussion. Thereafter, the participants shortly introduced themselves. To start the discussion, we asked participants to “think of an example of early screening for risks of mental health problems in healthy children due to environmental factors.” “Early screening” was defined as “identifying risks to children’s mental health before disease symptoms are experienced.” In this article, we refer to this as “presymptomatic screening” or simply “screening.” “Children” were defined as the ages zero to eighteen years old; the prenatal phases were excluded from the discussions. We deliberately refrained from providing examples or necessary aspects of screening to avoid influencing the responses and let the participants set the stage for the discussion from their own experience or imagination. We also refrained from querying about specific disorders, although participants were free to discuss those when brought up. To avoid group thinking, participants first wrote down their example individually, before sharing it with the group.

Subsequently, participants were asked to write down advantages and disadvantages of screening for risks for mental health problems in healthy children on separate sticky notes. A flipchart was used to organize the sticky notes and provide an overview for the discussion. Group discussion was stimulated by questions such as “why do you think this is a (dis)advantage?,” “does everyone agree?,” “what may be the impact on the child and his/her environment?” Thereafter, the same procedure was followed for discussing (dis)advantages of early screening for biological risk factors (biomarkers) for mental health problems in healthy children. Finally, as a concluding topic, participants discussed the question “to what extent do you think it is necessary and desirable to determine the risk of future mental health problems due to environmental factors in healthy children?” After the discussion ended, the moderator debriefed the participants with more information about the purpose of this study, its position within the larger Equal-life project, and more information about the Equal-life project.

### Data Collection and Analysis

The focus group discussions were audio recorded, transcribed, and translated into English when not conducted in the English language. For the development of a codebook, deductive and inductive coding was combined. For the analysis of the transcripts, the constant comparison method was used (Krueger and Casey 2015). First, two transcripts were independently coded in Atlas.ti 22.2 by SJ and SB using the protocol questions as a starting point to subdivide discussion parts under these main codes. The analysis focused on stated advantages and disadvantages of detection of mental health risks in general and for biomarker risk factors, reasons why participants considered these (dis)advantages important, and considerations about the desirability of detection of mental health risks in children. The two resulting codebooks were compared and discussed by all three authors to construct a codebook for the analysis of all transcripts, performed by SJ. In an iterative process, the codes and transcripts were constantly compared to each other, allowing for codes to be merged or assigned new labels. In parallel discussions among the three authors, codes were grouped into themes and subthemes until thematic stability was reached.

## Results

The focus group discussions had an open and informal atmosphere, without any participants dominating the discussions. After writing down their individual thoughts on sticky notes, participants discussed their viewpoints and experiences with screening for mental health risks. In most sessions, a large part of the time was spent discussing the promises and concerns of screening for mental health risks in general, sometimes limiting the time spent on biomarker screening.

When discussing mental health outcomes, participants often used general terms such as “mental health problem,” “neurological disorder,” and “psychiatric disease,” but in most discussions they also referred to specific disorders. Autism and ADHD were referred to in five separate discussions; anxiety and depressive disorders, eating disorders (e.g., anorexia), schizophrenia, and delays in developing speech and language were mentioned in three discussions. Furthermore, sleep disorders, personality disorders, intellectual disabilities, dyslexia, dyscalculia, drug addiction, and internet addiction were mentioned in one or two discussions.

At the beginning of the discussions, participants provided examples of two types of screening for risks to children’s mental health. The first type concerns methods of examining the child for risk factors. This includes questionnaires filled out by the children and/or their parents (dependent on the child’s age) and conversations with the child and/or parents. One participant provided an example of biomarker screening, i.e. to screen blood samples for lead concentrations. The second type concerns examining children’s social and physical environments. For example, by reviewing parental mental illnesses and stressors such as financial or relationship problems.

These examples set the stage for the further discussions about presymptomatic screening. In the following sections, we present the ethical considerations discussed in the focus groups. Our analysis revealed that these considerations could be captured under three main themes: 1) Promises and concerns about screening for risks to children’s mental health; 2) Additional considerations about biomarker screening; and 3) Implications for healthcare systems and society. In the introduction to each theme, we describe the central ethical considerations, which are then elaborated upon in the subthemes. See Table 1 for a summary of the themes, subthemes, and ethical considerations.

**Table 1:** Focus group themes, subthemes, and ethical considerations

Main themes	Subthemes	Ethical considerations
1. Promises and Concerns About Screening for Risks to Children’s Mental Health	1.1 Interventions to Prevent Mental Disorders 1.2 Concerns about Adverse Effects of Screening Practices 1.3 Careful Communication about Screening Provision and Outcomes	- The beneficial effects of screening need to outweigh the harmful effects - Screening outcomes should be actionable - Reliability and validity of screening tools - Stigmatization - Discrimination - Informed and autonomous decision-making
2. Additional Considerations About Biomarker Screening	2.1 The (Perceived) Objectiveness of Biomarkers 2.2 Biologization Concerns	- Validity of biomarker risk factors - Biologization of mental health - Medicalization
3. Implications for Healthcare Systems and Society	3.1 Screening Practices May Overburden Healthcare Systems 3.2 Exacerbating Society’s Disproportionate Focus on Risks 3.3 Unjustified Focus on the Child Instead of the Environment	- The population scale benefits of screening must outweigh the population scale harms - Justice: i.e. distributive justice and health equity - Stigmatization - Medicalization - Responsibilization

### Promises and Concerns About Screening for Risks to Children’s Mental Health

The participants considered mental health problems in children and adolescents and their social and economic impact on society an issue that deserves attention (e.g. increased healthcare costs, burden on healthcare and educational systems were discussed), and efforts should be made to tackle this issue. Participants demonstrated a strong concordance with the ethical principles of doing good and preventing harm (beneficence and non-maleficence). An important, related ethical consideration derived from their deliberations is the need for the potential benefits of screening to outweigh the potential harmful (psychosocial) effects. Furthermore, the reliability and validity of screening tools were considered important, as well as that screening outcomes are actionable. Participants indicated that mental illness is socially stigmatized and raised ethical considerations of stigmatization and discrimination as being potential adverse effects of screening, which may also provoke or increase other harmful effects. Finally, informed and autonomous decision-making was an important ethical consideration. Participants emphasized the need for careful and tailored communication about the aims, benefits, and risks of screening to promote children and parents’ informed and autonomous decision-making and improve the outcomes of screening practices.

It was, however, not obvious to the participants how to balance potential beneficial and harmful effects of screening among other things because children’s mental health and the context they develop and grow up in are highly complex. The main promises, concerns, and ethical considerations directly related to screening for mental health risks in children are discussed below and grouped into three subthemes: interventions to prevent mental disorders, concerns about adverse effects of screening practices, and careful communication about screening provision and outcomes.

#### Interventions to Prevent Mental Disorders

The main promise of screening for mental health risks discussed is that it provides opportunities for preventive actions to tackle the identified risks, diminish the risk of developing a disorder, or provide other kinds of support to at-risk children and families. This promises to reduce burdens and suffering from mental health disorders and their social and societal impacts. And when successfully identifying and supporting the most vulnerable children it promises to decrease health inequalities.

We would undertake this screening in the first months of life, and the literature states that the activities that will be undertaken in the first months will be reflected in the psycho-motor development even in the school period. So, all those children will have better academic achievements, better attention, and better concentration, so we would do it to implement an early intervention. (North Macedonia)

Participants agreed that “being early,” both in the developmental trajectories of disorders and early in life (i.e. the first one thousand days), provides the best opportunities to “be on time” to prevent disease and support children and families. For many participants, obtaining possibilities to act on the risk information, i.e. an action perspective, was an important condition for the acceptability of implementing screening programmes. These actions may be both preventing or reducing symptoms. These actions may occur within the medical domain, e.g. by offering behavioural programmes, but can also consist of other types of support such as structuring the child’s social and physical environment to minimize the risk of developing mental illness. The effectiveness of these preventive actions depends on many factors such as the type of risk, type of disorder, type of action, and willingness and opportunities of the child and parents to successfully implement the action.

Yes, identifies risks. Can be a benefit. And deployment before problems arise. For example, that ADHD, that you’re going to structure in advance, because structuring is good for your child, but for this child in particular. So, then you could structure in advance, that you can already deploy general tips. (The Netherlands)

#### Concerns about Adverse Effects of Screening Practices

Concerns were voiced that more knowledge of disease risks does not necessarily lead to better health outcomes but can also have harmful effects. A widely raised concern was the high complexity of mental health development. It is difficult to capture and predict the risk of developing most mental disorders as many different biological and environmental factors contribute to mental development. An additional complicating aspect is that most of these factors change over time. Furthermore, given the difficulty of accurately capturing risk factors in the social and physical environment in screening tools, participants warned for false-positive and false-negative results. When discussing biomarker screenings, some participants expressed scepticism about the reliability and validity of (future) screening tools that aim to capture the complex dimensions that shape mental health risks with biological data.

Participants discussed that screening outcomes can also have harmful psychosocial effects. For example, positive screening outcomes may cause worries and premature grief for a future with potential disease. Further concerns were raised that the identification of mental health risks may lead to overly focusing on the child’s mental health and the development of symptoms. This can harm the child–parent relationship. These adverse side effects need to be carefully considered, especially as there is always a possibility that the detected risks will not provoke the development of a disorder, even without preventive actions. Finally, information about mental health risks may even trigger a self-fulfilling prophecy as overly focusing on detected risks and corresponding adverse effects may increase the risk of disorders, something participants contrasted with screenings for physical health risks such as cancers.

I just think that in the psychological area you often have a self-fulfilling prophecy. Then you are sent there four times a year to the psychiatrist so with the question, and does he have it now, has something broken out? (Germany)

In addition, how people respond to screening outcomes is hard to predict. Some people may even start engaging in risky behaviours that increase the risk of disorder, as illustrated by this example of adolescents knowing they are at risk for psychosis:

For example, for risk for psychosis, and telling to adolescents, if you smoke pot, then you will get psychosis. Because, when you think of interest psychological processes, people are really illogical and adolescent might think “well, I’m going to get psychosis anyway,” so before. “I’m going to do everything I can. And I’m not going to go to school. I’d rather do homework” and, you know, all the rest. We can know, people are not that simple. When, and decision-making processes are very complicated and very unpredictable actually, so. (Slovenia)

Finally, stigmatization was mentioned in all focus groups as another important concern. Participants discussed that mental health disorders are socially stigmatized, adding a layer of sensitivity to discussing and implementing screenings for mental health risks. Children who are found to be at risk for mental illness may experience differences in how they are treated by peers, e.g. bullying, and by their parents and teachers, e.g. lower expectations of their learning capacities. Parents and children may also hide information or decide not to participate in screenings out of fear of stigmatization. Finally, participants were concerned that children at risk for mental illness may experience additional barriers or even discrimination in educational, occupational, and insurance contexts. For example, in the Stockholm focus group, barriers to joining the police force or the army were discussed:

And even a risk status could maybe already be a disadvantage in maybe, maybe it’s not that you’re now, but you cannot, but it might make it harder to?

Yeah. It’s like one step more to get there. So, it kind of depends also where you can go in life, you can set some barriers to maybe [...] you cannot go. (Dialogue between two participants in Sweden)

#### Careful Communication About Screening Provision and Outcomes

Besides the promises and concerns around offering screening practices and disclosing the outcomes to children and parents, participants discussed that the ways these outcomes are communicated can both help and harm the promises of screening programmes. Appropriate explanations by screening providers about the aims of a screening practice, what it is measuring, what the results mean, and the follow-up steps for different outcomes are required. As participants indicated that the difference between a risk factor and an (early) symptom, or between a risk for mental illness and being ill, is difficult to grasp for laypeople—and even for many health professionals—concerns were voiced that a positive result can be interpreted as a disease diagnosis or certainty that a disease will develop. This concern was even stronger when discussing biomarker screenings. However, careful and tailored communication between screening providers and children and parents was proposed to reduce the likelihood of misinterpretations on both sides. For example, when children or parents have difficulty understanding a questionnaire, additional explanations and support may increase the validity of its outcomes.

But I mentioned something about the fact that it is better not to separate the screening from the relationship and from a conversation, so that sometimes you first send out the screening questionnaire and only then do you have face-to-face contact. That you… maybe can go through the questionnaire together. And it’s not super-efficient, but then you might still get different information and different relationship and maybe faster access to parents—

Absolute.

—then if you say “I have read your questionnaire and suspect…

And sometimes even more honest answers.

Yes, I have also completed questionnaires together with parents, in which I think: ok, I see it very differently. (Dialogue between two participants in the Netherlands)

Therefore, in multiple focus groups, the recommendation was made that screening practices should always contain an element of conversation with the child and parents. Besides preventing misinterpretations, this allows for obtaining additional contextual information that may help assess the risk of developing mental disorders and determining suitable preventive actions. Finally, careful and appropriate communication may help the way screening outcomes are understood and acted upon by children and parents.

If you screen for something and then you have the, perhaps a health talk or something like that, a support talk to the parents, if I say it like that, then you’re talking about things and then you educate the parents because you tell them that this is very important, that this thing, the children need these things to be a good health people when they grow up, so you learn them things also. So, I think it’s a good way to have the talk also, not only by pointing out the things that’s not working well with this family, but also to tell them that this is very important to the children. (Sweden)

### Additional Considerations About Biomarker Screening

Most of the discussed promises and concerns were brought up for both non-biomarker and biomarker screening for risks to children’s mental health. This section presents promises, concerns, and ethical considerations that were discussed only for biomarker screening. Important ethical considerations identified are the need for highly valid biomarker screening tools and the avoidance of biologization and medicalization of mental health. To address these concerns, participants emphasized the importance of including social and environmental factors that also contribute to the development of mental disorders. In general, participants were more hesitant about the idea of deploying biomarker screenings compared to more traditional methods such as administering questionnaires or psychological evaluations. For example, in the Ljubljana focus group, the biomarker discussion started with strong statements on the undesirability of using biomarkers for screening, and the discussion remained mostly on disadvantages, even when asked for advantages.

I think it’s even worse if we have a biological marker then if we have a behavioural marker. (Slovenia)

The discussion in the Bremen focus group started with concerns for stigmatization and discrimination of persons at risk for mental illness, with several references made to German Nazi history throughout the discussion.

I don’t know, I don’t really like it at all, had a bit of resistance right away. (Germany)

On the other hand, the sentiment in the Skopje and Stockholm focus groups did not change when discussing biomarkers.

#### The (Perceived) Objectiveness of Biomarkers

Many participants remarked that biomarkers are generally considered to be more “objective” in comparison to the subjectiveness of psychological observations, questionnaires, and conversations. Measuring and depicting a biological marker was perceived as more straightforward and a biomarker may be easier to interpret compared to e.g. interpreting behaviours. For parents, this objectiveness can make it easier to accept risk information resulting from biological measures as participants expect them to perceive biomarkers as more reliable and truthful.

I think it’s an objective measure. I sometimes find it difficult, when do you call a child busy? What I find busy, you may not find busy. Whereas if you have a certain cortisol level in your blood or something, that might be a much clearer point to say something about.

You objectify it, yes.

Well, it’s a little different I guess, that’s more that it would be a clearer signal for parents.

Yes, a kind of recognition huh, confirmation.

I see I had exactly the same thing here, indeed a kind of proof for parents.

That may be for professionals more …

Also, maybe for parents. Because to me it’s just a busy child, but apparently, it’s ADHD, because we crossed a line. (dialogue between three participants in the Netherlands)

However, the perceived objectivity was also labelled as misleading and evoking even stronger assumptions of disease development being inevitable, feeding into the harmful psychosocial effects described in Sect."Concerns about Adverse Effects of Screening Practices".

I had the opposite from my advantage was that, that these could be, like experience as a fact and truth, you know. So, but then the person like over-interpreted thing and thinking that this is the only truth and ignoring other perspectives. So, like somebody could be like “this is, it says, it’s hard fact, it’s like numbers and it’s a test, this is the truth,” you know.

And it’s different from having a questionnaire outcome.

Yeah. That could never be like fact in the same way. (Dialogue between two participants in Sweden)

#### Biologization Concerns

Another concern was that the biological nature of the risk factors may cause a focus or preference for biological explanations and solutions, both in practitioners and parents, especially as participants noted that physical illness is in general more societally accepted compared to mental illness. The societal stigma that rests on mental illness may make parents more easily accept biological solutions such as medications, while psychological help may be refused although it still is potentially beneficial. Biologization—the reduction of mental health to a purely biological phenomenon—was also seen as possibly neglecting important contextual factors and beneficial preventive actions to improve a child’s context.

So, it’s nice to hear that there’s something there, okay, so there’s also something that can be done to prevent it or to take it away. That you are very quickly inclined to do that. Find and fix. So can I also do something physical. And so do we also have to fix it that way and have to put a pill in. (The Netherlands)

Overall, participants felt that if mental health biomarker screening tests become a possibility, they should not be used independently but always be combined with psychological assessments in the form of conversations and/or questionnaires. This was thought to provide a better understanding of the individual child and his/her environment and reduce concerns about biologization. However, doubts were also expressed about the added value of biomarkers, as some participants expected it to not provide additional useful information over non-biomarker screening practices.

### Implications for Healthcare Systems and Society

In all focus groups, the discussions went beyond discussing the promises and concerns of screening practices, to discussing their embedding in healthcare systems and society. Various strategies to promote public health were discussed, taking into account multiple ethical considerations, including justice considerations such as the fair allocation of resources (distributive justice) and increasing health equity by supporting vulnerable populations. Overall, the public health benefits of screening must outweigh the potential harms at the population level, such as stigmatization of subpopulations and overburdening the healthcare system. Other important ethical considerations were that the focus on risk factors rather than beneficial factors for mental health may reinforce medicalization and that a lack of integration of the child’s social and environmental context in screening risks placing causes, solutions, and responsibilities in individual children and parents. Therefore, it is also important to consider how improving social welfare and shared environments can support children’s well-being and development. We have grouped these wider implications into three subthemes: screening practices may overburden healthcare systems, exacerbating society’s disproportionate focus on risks, and unjustified focus on the child instead of the environment.

#### Screening Practices May Overburden Healthcare Systems

Participants discussed how preventive actions may prevent the development of mental illness and reduce the burden on the healthcare system as fewer children need specialized medical treatments. A distinction was made between universal and targeted screening programmes. The latter was proposed to increase health equity by targeting the most vulnerable populations, but labelling certain groups also risks evoking stigmatization. Universal programmes can avoid stigmatization, but participants worried that screening all children will identify a high number of at-risk children who require further medical examinations. This would require many healthcare resources, possibly opposing the detection and support of the most vulnerable children and provoking overdiagnosis and medicalization.

… the [universal] screening, you will actually lose the capacity to identify the most vulnerable. Because you will get such a, such a large number of people, who have mental health problems. (Slovenia)

Besides, several participants noted that the healthcare systems in their countries already have a shortage of psychologists and other healthcare professionals. Thus, an important precondition for the implementation of screening programmes is a healthcare system with sufficient capacity to perform screening activities and follow-up procedures.

… you have the issue of capacity of the system, how many people you get in the system? What do you do? What kind of diagnostics you have in place? What kind of treatment options you have in place? (Slovenia)

#### Exacerbating Society’s Disproportionate Focus on Risks

In multiple focus groups participants voiced a broad critique of the excessive focus on risks they perceive in healthcare and society at large. They noticed that meanings of mental health and illness are shifting such that more and more behaviours are considered undesirable and unhealthy. Screening programmes may reinforce this “risk orientation,” especially as screening programmes often do not assess protective salutary factors and participants denote a lack of attention to children’s resilient potentials.

Maybe, on a societal level, it has effects of changing like this lens, looking for risks rather than positive factors, it’s something to consider.

Yeah, because you become a bit more helpless, if you only look at the negatives and then the risks and, you know, maybe feeling like there’s nothing I can do, and nobody can do anything, instead of seeing those as well, but also seeing the strengths and the positives and then you can maybe feel more, like, I can do something, I can change it, I can do more of these things. So, at least, like mentally, the focus could have an effect on how you, you know, live your life. (dialogue between 2 participants in Sweden)

Participants noted that in defining risks to healthy development, more and stricter standards are set for “normal” versus “deviant” development and they argued that a stronger orientation towards risks can lead to an unnecessary problematization and medicalization of children’s behaviours and developments. Participants also cautioned that these standards may leave little room for diversity of personalities and developmental trajectories. Participants assigned value to giving children space and time to follow their own developmental trajectories and noted that mental deviations can also produce or be accompanied by valuable mental features such as creativity. This was found to potentially conflict with the wish to find risks as early as possible to have time to perform preventive actions to “correct” a child’s development.

… but to what extent is something normal, is it normal behaviour or is it deviant behaviour. And to sort of … there you are also in the normalization of a child with ADHD, or a child of six who just finds it very difficult to sit still all day on a chair. What are we talking about? (The Netherlands)

#### Unjustified Focus on the Child Instead of the Environment

Concerns were expressed that this orientation to health risks in combination with screening practices that aim to identify individual (biological) risks in children leads to a general disregard of more structural external environmental factors. Participants discussed that screening programmes often do not pay (enough) attention to the context a child is growing up in, risking placing the causes, solutions, and responsibilities in the child itself.

Exactly, also because there is the idea that the problem, that is, the screening is on the child who has the problem, the discomfort. Actually, the child’s problem is often a family problem, it’s a problem of the parent failing, of the fatigue of the context. Then, clearly, the child presents a discomfort and you have to intervene there, but if you don't intervene with those who are with that child, that is at the level of prevention also—If you work only with the child and you don't work with the family, then it is difficult. (Italy)

This raised concerns about causing children or parents to be found “guilty” of detected risk factors while circumstances such as poverty or abuse are often outside their control.

So, about a lot of things I think, we are so specialized in a certain area to find things out and also to treat for my sake, but the social factors are not changed. So many children would not be sent for diagnostics because of behavioural disorders or suspected autism spectrum disorders if there were a good care situation with a much better staff ratio, I am convinced of that. And so, we are sharpening our instruments in order, how should I put it, to somehow categorize individual, conspicuous behaviour. But we don’t change anything about the conditions under which it arises socially. (Germany)

Family and school environments were discussed as relevant contexts for children’s mental development. It was proposed that screening programmes should include these contexts in their risk assessments, or even take place within these contexts, and that preventive actions within these environments can be effective, e.g. adapting educational programmes to support children at risk. When implemented in schools, screening programmes may also be used within a wider movement to normalize mental health issues and make them easier to discuss, possibly contributing to reducing mental health shame and stigma.

The importance of taking into account the child’s environment led some participants to suggest that screening and targeted implementation of preventive actions for mental health risks might not be the most effective way of preventing mental health disorders and promoting healthy mental development. Improving the environments in which children grow up can be more effective to promote public health and less stigmatizing. Implementing or expanding social welfare policies for all children and families, with or without the need to screen them for risks first, was proposed.

Because the first answer are population-based interventions, that are prevented in the first level. And it’s not always, that you always need a psychologist. You need housing and food, good friends, good neighbours. (Slovenia)

Realizing the societal foundations for “good care situations” for all children was believed to prevent a wide range of possible health issues and reduce the need for screenings and might also be more cost-effective in the long run. Participants proposed, for example, educational programmes for children and parents to stimulate discussions on mental health, supporting schools to provide healthy and stimulating school environments for all children and programmes for parents with mental health or financial problems.

But of course, there are some general interventions like parenthood training, and this can be very general. It doesn’t need any special screening and it’s really helping. (Slovenia)

## Discussion

This focus group study aimed, as the first of its kind, to explore child mental health professionals’ views on presymptomatic screening for mental health risks in children. We performed six focus groups, in different European countries, with in total thirty-four professionals from the child mental health domain. This resulted in in-depth insights into these stakeholders’ views on presymptomatic screening for risks to mental health in children. Identified ethical considerations included the benefits of screening need to outweigh the harms (at the individual and population level), the reliability and validity of risk factors and screening tools, informed and autonomous decision-making, screening outcomes need to be actionable, stigmatization, discrimination, biologization, medicalization, responsibilization, and justice considerations. Here, we reflect on the identified ethical considerations and situate our findings within the wider ethical debate on presymptomatic screening for risks to children’s mental health and provide recommendations for future research and practice.

The results show that both the interests of individual children and parents as well as collective societal interests are important ethical considerations. Participants did not explicitly balance or contrast these different interests but rather discussed them side-by-side. This is comparable to the ethics literature on presymptomatic screening where these ethical considerations are typically discussed alongside rather than in explicit opposition or balance (Jansen et al. 2024). Within the broader public health ethics literature, this issue is traditionally framed as a tension or balancing between promoting the health and well-being of the population and safeguarding the rights, autonomy, and freedoms of individuals (Kass 2001). This is often referred to as the proportionality principle, i.e. the expected public health benefits should outweigh the possible harms to individual rights and well-being (Childress et al. 2002).

Ethical considerations on the benefits of presymptomatic screening for individual children mostly relate to providing opportunities for timely preventive actions to tackle the identified risks, diminish the risks, or provide other kinds of support to at-risk children and families. Within the ethics literature, the potential of biomarker approaches to help predict the risk of developing a particular psychiatric disorder before symptoms appear and to prevent and timely treat these disorders are also considered as promising. These promises can, however, only be fulfilled if preventive or supportive interventions are available and can be implemented (Singh and Rose 2009), which was also discussed in the focus groups. Our participants expressed uncertainty about whether these conditions are present in current healthcare systems (see Sect. "Screening Practices May Overburden Healthcare Systems"), making this an important ethical consideration. Moreover, the feasibility and desirability of presymptomatic screening were often questioned.

With regards to the feasibility of accurately predicting mental health outcomes, participants were concerned about the high complexity of capturing and predicting mental disorders, as mental health is shaped by complex and dynamic biological and environmental factors. These concerns relate to ethical considerations about the need to adequately understand how a disease develops, what determinants are important for its development, and the reliability and validity of screening methods to measure these factors. This is in line with concerns in the literature about the low predictive value of risks and low translational value to individual cases (Singh and Rose 2009; Thomas 2015). Research efforts to incorporate higher levels of complexity may increase the accuracy of predictions. For example, research into the exposome, i.e. the totality of internal and external physical and social environmental exposures over a lifetime, offers a new framework to study the complex interactions between exposures and individual characteristics that determine (mental) health risks and their underlying disease mechanisms (Kamp et al. 2022; Wild 2005). In the focus groups, no remarks were made about these scientific developments, suggesting a gap between research and practice. Educating mental health professionals about these developments could help bridge this gap and prepare them for future practices. Nevertheless, increasing the amount of data included in screening practices will not solve all issues in capturing the complexity of mental disorders. Definitions and classifications for many disorders remain disputed and sensitive to societal and cultural shifts (Clark et al. 2017; Jablensky 2016).

Participants brought forward ethical considerations about how defining and detecting risks for mental disorders might result in stigmatization. However, we observed that they had difficulty explaining exactly what their concerns for mental health stigma meant and how stigmatization would be caused by screening. The literature on mental health stigma distinguishes different types, impacts, and explanatory mechanisms of stigma. A distinction is often made between public or social stigma and self-stigma. Public stigma consists of a large social group endorsing negative beliefs about, and acting against, a designated group. Self-stigma occurs when a person internalizes these negative beliefs, often causing a loss of self-esteem and self-efficacy (Corrigan et al. 2005; Corrigan and Watson 2002; Spriggs et al. 2008). In the focus groups, the existence of public mental health stigma was presented as an undisputed fact. This was discussed to have harmful effects such as parents and children avoiding participation in screening or withholding information relevant to a screening. However, no clear references to self-stigma were made. Self-stigma might indeed be less of a problem in the case of presymptomatic screening as it only indicates a risk and not a diagnosis. However, given concerns about people’s difficulty to distinguish between being at risk and being ill, both for biomarker and non-biomarker screening, self-stigma cannot be ruled out and is worth investigating further.

Overlap in ethical considerations for both biomarker and non-biomarker screening was found for most discussion topics. The literature generally presents presymptomatic biomarker screening as a novel medical innovation that raises unique social and ethical concerns due to its innovative nature and promise of greater prediction accuracy (Hoge and Appelbaum 2012; Glenn 2019; Singh and Rose 2009). Our participants recognized that the biological nature of screening tests and risk factors are distinct features that provoke considerations such as (the perception of) being objective and pushing for biological explanations and solutions. Although these features are unique to biomarker screening, the concerns they provoked in the focus group discussions overlapped with those raised in relation to non-biomarker screening. For example, both were expected to evoke assumptions of disease development being inevitable, although this effect was thought to be stronger in biomarker screenings. In addition, throughout both discussions concerns were brought up about how screening would locate explanations and solutions within the child itself rather than considering important contextual risk factors and solutions. The overlap in concerns between biomarker and non-biomarker screening that we observed can be interpreted in two ways. First, it could be the case that for mental healthcare and policy, biomarker and non-biomarker screening, despite underlying differences in methods, effectively raise the same concerns in practice. If so, these findings point to a need for developing a more unified understanding of the social and ethical implications of general presymptomatic screening to accommodate the practice reality. Second, an alternative interpretation is that participants’ unfamiliarity with biomarker screening practices may have influenced the direction of the discussions. If so, participants might not have been able to identify promises and concerns unique to biomarker screening. This would point to a need for communication of mental health biomarker developments and educational programmes to prepare healthcare professionals for presymptomatic screening applications. Further research is required to resolve this ambiguity.

The ethical consideration of privacy is widely discussed in screening literature but was not found to be discussed considerably in these focus groups. Furthermore, although the participants often alluded to the importance of adequate communication and information provision about the aims, benefits, and risks of a screening programme, the practice of obtaining informed consent and providing information about e.g. the confidentiality of test results was not discussed (Ahlgrim et al. 2019; Corcoran et al. 2005; Spriggs et al. 2008; Thomas 2015). Mental health professionals may be unaware of these topics or do not consider them as important as the topics brought forward. Another explanation may be that privacy regulations and informed consent procedures are already deeply ingrained into mental health practices and policies, making them no longer a concern to participants. Notwithstanding, the expressed uncertainties about the balance of benefits and harms raise important questions for informed consent procedures. For instance, how can healthcare professionals convey these benefits and harms of presymptomatic screening to parents and children? Given professionals’ uncertainties, can parents reasonably be expected to make informed decisions about their child’s participation in presymptomatic screening? To what extent can professionals and parents decide about a child’s best interests and how to weigh this against the right to an open future? These uncertainties are reason for caution about promoting presymptomatic screening for mental health risks, and one might even consider refraining from offering pro-active screening until a positive balance between benefits and harms is beyond doubt.

Besides the need for balancing benefits and risks for individual families, methods, and disorders, the findings show that presymptomatic screening poses ethical considerations regarding how to safeguard the broader societal interests as well. One such dilemma concerns the allocation of limited resources. On the one hand, the high burden of mental illness on children and adolescents and the corresponding burden on collective healthcare resources and society support allocating resources to the development and implementation of preventive programmes. However, participants cautioned that screening will initially increase the burden on healthcare systems, which is a problem given current shortages of staff and resources. It may even divert resources from children who are most vulnerable and in immediate need of care now. Furthermore, our focus group study underlines the importance of taking the child and family’s larger environment and social context into account in risk assessments. Participants expressed hesitation about the capability of screening methods to do so and suggested that existing interventions such as educational programmes and population-wide social welfare interventions may be more effective in addressing these complexities. Policymakers are called to carefully deliberate the various options for promoting children’s mental health and strike a balance between the proposed long-term benefits of prevention programmes and the pressing demands of the current healthcare system.

This study is not without limitations. First, its inclusion of European-based child mental health professionals limits its generalizability tot non-European contexts, where cultural and societal values may differ significantly. For instance, patient autonomy holds varying importance across different regional contexts, and mental health stigmatization is shaped by an interplay of cultural, societal, and normative factors. Although this variability likely exists within the included six countries as well, a comparison between the included countries was out of the scope of this study. Second, the translation of discussions to English may have resulted in loss of nuances and subtleties in the participants’ views. Third, the method’s emphasis on allowing participants to engage in the discussions from their own experiences and imaginations may have unintendedly overlooked certain topics, such as privacy, that did not naturally arise. Furthermore, the selection of participants based on their experiences in the child mental health practice, rather than inviting experts in ethics, necessitated the authors to engage in a secondary analytical step to identify the ethical considerations from the discussions.

This study’s findings also raise fundamental questions about our normative assumptions regarding what behaviour is normal and healthy and about what conditions society should secure for a healthy development of children. These questions extend to the moral obligations and responsibilities assigned to different stakeholders—including parents, governments, institutions, and businesses—to mitigate the various biological, environmental, and social risk factors to protect and promote the well-being of children. The ethical considerations identified in this study can be applied to specific case scenarios, such as specific disorders, target populations, and screening methods, to help assess their ethical justifiability and inform their development, implementation, and evaluation.

## Conclusions

This study utilized focus group discussions to investigate the views of mental health professionals about presymptomatic screening for risks to children’s mental health. Identified ethical considerations included the benefits of screening needing to outweigh the harms (at the individual and population level), the reliability and validity of risk factors and screening tools, informed and autonomous decision making, the actionability of screening, stigmatization, discrimination, biologization, medicalization, responsibilization, and justice considerations. Our findings underscore the necessity of exercising caution in the development and potential implementation of presymptomatic screening for mental health risks in children: for both individuals and society, benefits of screening should clearly outweigh the potential harms.

## Data Availability

The transcript data are not publicly available as they contain information that could compromise participants’ privacy and consent.
